# Use of Unmanned Surface Vehicles (USVs) in Water Chemistry Studies

**DOI:** 10.3390/s24092809

**Published:** 2024-04-28

**Authors:** Georgios Katsouras, Elias Dimitriou, Sotirios Karavoltsos, Stylianos Samios, Aikaterini Sakellari, Angeliki Mentzafou, Nikolaos Tsalas, Michael Scoullos

**Affiliations:** 1 Athens Water and Sewerage Company S.A. (E.Y.D.A.P.)-Research and Development, Oropou 156, 11146 Athens, Greece; samios@eydap.gr (S.S.); ntsal@eydap.gr (N.T.); 2 Institute of Marine Biological Resources and Inland Waters (IMBRIW), Hellenic Centre for Marine Research (HCMR), 46.7 km Athens-Sounio Ave., 19013 Anavyssos, Greece; elias@hcmr.gr (E.D.); angment@hcmr.gr (A.M.); 3 Laboratory of Environmental Chemistry, Department of Chemistry, National and Kapodistrian University of Athens, Panepistimiopolis, 15784 Athens, Greece; skarav@chem.uoa.gr (S.K.); esakel@chem.uoa.gr (A.S.); scoullos@chem.uoa.gr (M.S.)

**Keywords:** remote sensing, water quality, autonomous USV, inland and coastal waters, telemetric stations

## Abstract

Unmanned surface vehicles (USVs) equipped with integrated sensors are a tool valuable to several monitoring strategies, offering enhanced temporal and spatial coverage over specific timeframes, allowing for targeted examination of sites or events of interest. The elaboration of environmental monitoring programs has relied so far on periodic spot sampling at specific locations, followed by laboratory analysis, aiming at the evaluation of water quality at a catchment scale. For this purpose, automatic telemetric stations for specific parameters have been installed by the Institute of Marine Biological Resources and Inland Waters of Hellenic Centre for Marine Research (IMBRIW-HCMR) within several Greek rivers and lakes, providing continuous and temporal monitoring possibilities. In the present work, USVs were deployed by the Athens Water and Sewerage Company (EYDAP) as a cost-effective tool for the environmental monitoring of surface water bodies of interest, with emphasis on the spatial fluctuations of chlorophyll α, electrical conductivity, dissolved oxygen and pH, observed in Koumoundourou Lake and the rivers Acheloos, Asopos and Kifissos. The effectiveness of an innovative heavy metal (HM) system installed in the USV for the in situ measurements of copper and lead was also evaluated herewith. The results obtained demonstrate the advantages of USVs, setting the base for their application in real-time monitoring of chemical parameters including metals. Simultaneously, the requirements for accuracy and sensitivity improvement of HM sensors were noted, in order to permit full exploitation of USVs’ capacities.

## 1. Introduction

It is crucial for water catchments to maintain their water quality conditions and strive, if possible, to achieve a high ecological and chemical status by the year 2027 [1,2,3]. In recent decades, catchments have gained significant attention in global water policy discussions. Integrated Water Resources Management (IWRM) is increasingly acknowledged as a holistic approach [4,5,6], leading to the development of the Integrated Lake Basin Management (ILBM) to address basin-specific characteristics. Evaluating the effectiveness of catchment basin management schemes and practices is essential [7,8,9].

Strategies for monitoring water quality are vulnerable to the irregular and unpredictable nature of precipitation events and hazardous incidents, such as sewage discharge, combined sewer overflows and agricultural runoff, which can impact drinking water resources and recreational water bodies [8,10]. These events occur sporadically and are often not captured by traditional monitoring approaches, which are characterized by limitations in terms of their temporal and spatial scope [11,12]. Such limitations hinder the assessment of water-related pressures and the development of effective countermeasures [13]. While automatic monitoring stations offer the potential for near real-time monitoring of basic water quantity and quality parameters, they are constrained by their point measurement nature, requiring significant investments to achieve the desired spatial resolution [14].

The need for an enhanced monitoring strategy yielding complete datasets with higher spatial resolution has become critical, requiring an approach which would enable the prediction of water quality at the catchment scale and pave the way for its improvement [15,16,17,18,19]. Nowadays, innovative genetic algorithm-based equations can be employed as indices for comparing with observed data, yielding more realistic results compared to experimental data [20,21]. Another valuable tool is the application of satellite data, which provides high temporal and spatial coverage, although it is subject to specific timescales and parameters (e.g., chlorophyll α) with limitations based on weather conditions [22,23]. An innovative approach gaining rapid traction in water monitoring is the utilization of unmanned surface vehicles (USVs) [10,17,18,19,23,24,25,26]. Although several commercial USVs are already on the market, ongoing research remains dedicated to advancing the technology and uncovering novel applications for these vehicles.

In the framework of the Horizon 2020 INTCATCH project (G.A. No 689341), a range of USVs has been developed and demonstrated throughout Europe [26,27,28,29,30]. These prototypes of short-length USVs that can be controlled remotely or can use an autonomous sailing mode are equipped with innovative commercial systems providing valuable data on various aspects of water quality. The main parameters are physical such as electrical conductivity and temperature, chemical such as dissolved oxygen, pH and metals, as well as biological such as chlorophyll α. All those parameters constitute variable ecological quality elements both spatially and temporally, the reliable assessment of which requires the availability of multi-year monitoring data. USVs’ application eliminates the need for labor-intensive and expensive monitoring methods, assuring more efficient processes.

A more comprehensive evaluation of the water quality of rivers and lakes in Greece might become feasible through the combination of existing monitoring strategies with innovative technological tools such as USVs. The study areas comprise Lake Koumoundourou, a lagoon situated near the industrial zone of Athens, along with three rivers—Acheloos, Kifissos and Asopos—which are typical sites for investigating catchments in Greece. These areas are subject to agricultural, urban and industrial pressures, each exhibiting fluctuations of distinct physicochemical characteristics. These water bodies likely play crucial roles in the local ecosystems and communities they serve. They are characterized by a diverse nature and dynamics, facing several key anthropogenic pressures derived by industrial activities, extensive livestock farming in sub-basins draining into lakes or rivers and agricultural practices [31]. Additional activities potentially affecting water resources comprise fisheries, hydropower, recreation and cultural practices within the catchment area, including water abstraction for irrigation and drinking purposes [32]. These anthropogenic pressures also encompass various water quality challenges related to eutrophication and thus to occasional algae blooms [10,33].

The main objective of the present work is to demonstrate both the advantages and disadvantages of employing innovative and integrated USVs alongside telemetric stations within existing monitoring strategies for rivers and lakes in Greece, in order to achieve a more comprehensive evaluation of their water quality. For this purpose, the use of advanced technologies like USVs can provide valuable insight into the ecological status of these aquatic environments. Given the diverse nature of the study area—incorporating both a lake and multiple rivers—it is likely that data collected from USVs and potentially integrated with telemetric stations would offer a comprehensive understanding of water quality dynamics across different water bodies. This comprehensive approach enables the identification of specific issues, trends or variations in water quality parameters across these varied environments.

During demonstrations in Greece, significant points emerged regarding the exploitation of USV services, particularly those of interest to end-users. Notably, the use of USVs coupled with online sensors for water monitoring is a novel technology not only in Greece but globally, sparking interest among stakeholders and monitoring organizations. As sensor technology advances, the potential applications of USVs expand, with particular interest for monitoring and sampling in inland waters. Positive perspectives include the fast and flexible data collection, safe and easy data organization and storage, convenient access to sampling areas with limited accessibility, improved management of site sampling plans, provision of spatially distributed data and the creation of a safer working environment compared to typical survey methodologies. However, while USVs offer significant advantages, they also come with limitations. These may include the need for enhancements in Artificial Intelligence (AI) and Internet of Things (IoT) systems, the high cost of specialized sensors, the requisite level of expertise for proper operation, the necessity of sensor calibration before each use, and the time-consuming preparation prior to each sampling campaign. Additionally, improving sensors and electrodes, particularly in terms of selectivity, could further enhance the effectiveness of these vessels.

## 2. Materials and Methods

### 2.1. Unmanned Surface Vehicles’ Operation and Description

**Figure 1:**
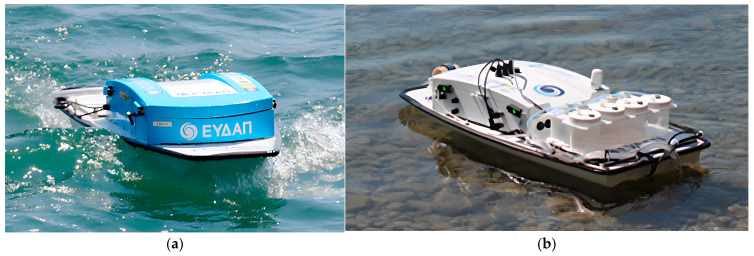
Two prototype USVs: (**a**) Chlα and (**b**) HM and Sampling, both equipped with the basic sensor Kit (DO, T, pH and EC).

The Athens Water and Sewerage Company S.A (Athens, Greece) has been responsible since 2019 for the introduction and validation of USVs application within Greek catchments into the framework of Horizon 2020 INTCATCH project [10,23]. The system structure consists of an unmanned surface vehicle of approximately one meter in length (Figure 1) and several electronic components facilitating boat control and sensor data acquisition. The USV has a low profile to minimize wind resistance and can be deployed in as little as 25 cm of water, either operated manually using a remote control (RC) device with 500 m range or controlled via a tablet, which provides high-level instructions such as monitoring of a specific predefined area using autonomous navigation [26,27]. GO-Systemelektronic (GO-SYS) provided the electronic components that are housed within the boat. A BlueBox is responsible for overseeing the sensors and circuit boards [28]. It locally stores sensor data in a flash memory and transmits it to the Raspberry via a serial connection. The sensors are mounted to a two-piece frame, protecting them from damage and facilitating horizontal attachment to the underside of the USV. A lithium polymer (LiPo) battery, located in the back compartment of the USV, provides the necessary energy for continuous operation for up to 3 h. Data collected are streamed in real-time to a cloud-based information system [28] and then can be downloaded or accessed through the web-based application WAter QUality INspector (WAQUIN) for visualization [30]. During the data collection campaign, a mobile application [30] allows for real-time visualization of the data, presenting the results obtained using the specific color patterns of blue, green, yellow, orange and red, complying with an increasing values scale. All USV prototypes were equipped with commercially appropriate sensors from GO-SYS for the monitoring of standard parameters such as dissolved oxygen (DO), temperature (T), pH and electrical conductivity (EC). The integrated system of chemical parameters (pH, EC, T and DO) on the USVs was thoroughly validated at University of Natural Resources and Life Sciences in Vienna (BOKU) [29]. Moreover, it should be underlined that the measurements by the USVs’ sensors were in very good agreement with parallel measurements performed in the field with portable devices and in the EYDAP laboratory with standard analytical methods. It is noteworthy to mention that the USVs can be seamlessly integrated with other commercial sensors (e.g., depth sensor) and even cameras. In this work, constant visual observation of the study area was essential, highlighting the versatility and adaptability of USVs in environmental monitoring applications. Among the INTCATCH USVs, two were equipped with specialized sensors: one for chlorophyll α (Chlα) and the other for heavy metals (HMs). These autonomous USVs, namely the ‘Chlα’ USV and ‘HM and Sampling’ USV were employed herewith for water chemistry studies. The ‘HM and Sampling’ USV, in particular, was equipped with an autonomous sampling system designed for collecting water samples for testing in the laboratory.

‘Chlα’ USV: This USV (Figure 1a) is equipped with a Turner Designs CYCLOPS-7 Submersible Fluorometer/Turbidimeter, an accurate single channel detector measuring Chlα with a detection limit of 0.03 μg L^−1^. A set of three additional probes provides measurements of DO at a range of 0–20 mg L^−1^ with a resolution of 0.1 mg L^−1^ and a measuring frequency of ≥1 s, T at a range of 0–50 °C, pH with a resolution of 0.05 and EC at a range of 40–4000 μS cm^−1^ and a resolution of 1 μS cm^−1^.

‘HM and Sampling’ USV: Besides the basic set of probes measuring standard parameters (DO, T, pH and EC), this USV (Figure 1b) is equipped with an easy-to-use system for Pb and Cu measurement via the Square Wave Anodic Stripping Voltammetry (SWASV) method, and also a sampling system enabling the collection of four (4) different water samples of 0.5 L each using peristaltic pumps.

### 2.2. Hydro-Telemetric Stations

**Figure 2:**
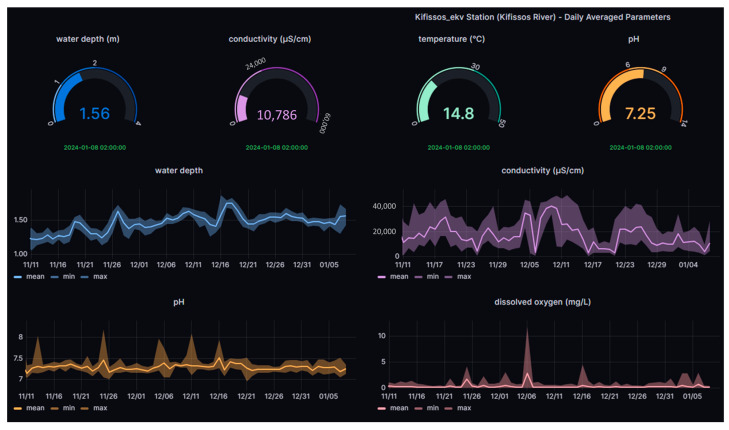
Dynamic real-time visualization platform.

The Institute of Marine Biological Resources and Inland Waters (IMBRIW) of the Hellenic Centre for Marine Research (HCMR) has established an extended network of automatic telemetric stations across various lakes and rivers in Greece [34]. Stations situated at Koumoundourou Lake and Kifissos estuaries (Table 1, Figure 2) provide real-time data at an hourly basis for parameters including water level, pH, EC, T, DO and Oxidation-Reduction Potential (ORP). At the telemetric station within Koumoundourou Lake, salinity measurements are also recorded together with the daily averaged quality index based on DO [35] and the Canadian Council of Ministers of the Environment Water Quality Index (CCMEWQI) [36] fluctuation. 

### 2.3. Metals System

The low-cost fluidic innovative metals system is based on the method of Square Wave Anodic Stripping Voltammetry (SW-ASV) for the determination of lead (Pb) and copper (Cu). The performance characteristics of the measuring system installed on the USV for the analysis of Pb and Cu were obtained under controlled laboratory conditions, utilizing a calibration curve prepared with mixed solutions of Cu and Pb, with a Pb:Cu ratio 1:4 [37]. The limits of detection (LOD) were estimated to be equal to 4 and 7 µg L^−1^ for Pb and Cu, respectively. De Vito-Francesco et al. (2022) was aware that the limit of quantification (LOQ) values of the metals system [38] were not low enough to address the limits of the environmental quality standards (EQS) established by the Water Framework Directive (WFD) [39]. On the other hand, results indicate that the developed integrated metals system is a promising tool for investigative monitoring according to the WFD, i.e., to ascertain the impact of urban and/or accidental pollution sources in inland surface waters [38]. Measurement precision levels, evaluated as reproducibility and given as relative standard deviation (RSD in %), were 11–18% and 6–10% for Pb and Cu, respectively, which is considered satisfactory for on-site measuring systems [38].

The Square Wave Anodic Stripping Voltammetry Heavy Metal System operates following several sequential steps [37]:

1. Conditioning Step: The working electrode is subjected to a positive potential (or at least 0.0 V) to prepare for the subsequent steps with a deposition time of 180 s.
2. Deposition Step: A deposition potential is applied to the working electrode, causing the reduction and deposition of certain metals or other species present in the sample onto the electrode surface. The deposition process occurs based on mass transport, and typically occurs at a thin interface between the sample and electrode with a potential from −1.2 to −1 V [37] for 200 s. In flow-through mode or under stirring conditions, an increase in the signal can be observed.
3. Equilibration Step: This step allows for the equalization of ion concentrations within the boundaries of the electrode surface, ensuring uniformity prior to proceeding to the next step. The equilibrium step is applied for 20 s, while the peristaltic pump is stopped.
4. Stripping Step: Metals previously reduced and deposited onto the electrode during the deposition step are now released (oxidized) by applying a square wave potential within a specific range. Each metal undergoes oxidation at a specific potential value, resulting in the recording of various peaks at different potentials. The electrical current measured during this process is proportional to the concentration of the metals. Metal concentrations in the sample solution are determined through the interpretation of the voltammograms obtained using appropriate software. The peak height or area is compared to standard concentrations for calculation purposes.

To perform the above method, a metals measuring system is required, including a potentiostat, a sensing electrode system (screen-printed sensors), a flow cell, a peristaltic pump, software and a data processing system [37,38].

An electrochemical analytical system was developed and optimized exclusively in the framework of the INTCATCH project for detecting heavy metals in surface water [38]. The system consists of a miniaturized sensing device integrated into a microfluidic flow cell, which is part of a flow system including a pump, a gas bubble trap and a data acquisition device. Initial tests were conducted using synthetic water samples containing cadmium, copper, lead and zinc, in order to evaluate the capabilities of this technology.

Following its performance evaluation with real water samples in the laboratory, the device was successfully integrated into the ‘HM and Sampling’ USV, creating an autonomous and remote-controlled system capable of detecting lead and copper in surface waters and simultaneously collecting water samples. This integrated system underwent validation through indoor and outdoor experiments at BOKU and was provided to the INTCATCH end-users for further validation and use [38].

### 2.4. Study Area and Data Collection

**Figure 3:**
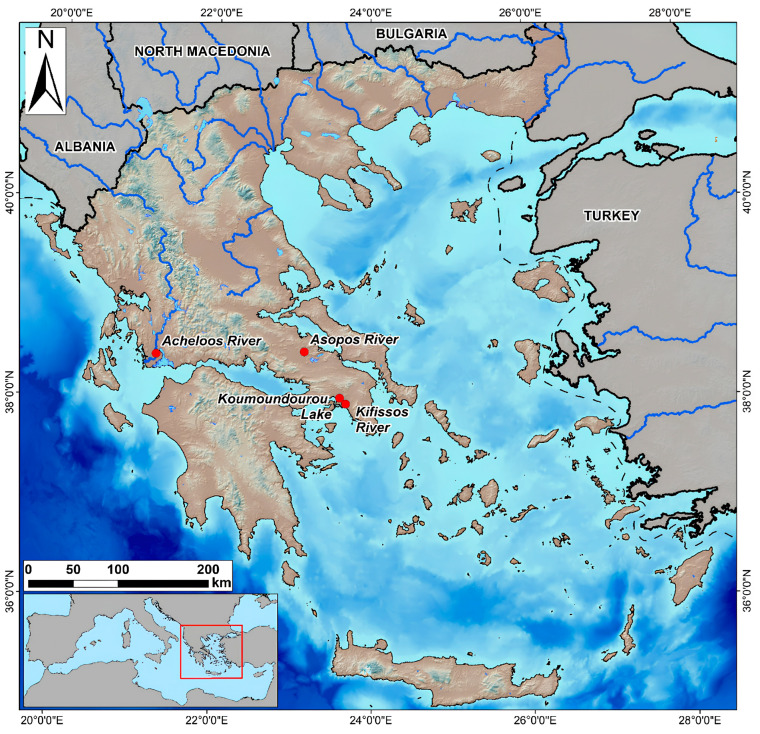
Study areas in Greece with the use of USVs.

Introducing an innovative water quality monitoring strategy in Greece using USVs for the first time in 2019 is a significant step toward enhancing environmental monitoring practices in the country. The study area comprising Koumoundourou Lake and three rivers—Acheloos, Asopos and Kifissos—holds immense importance in understanding and managing Greece’s water resources (Figure 3).

#### 2.4.1. Koumoundourou Lake

Koumoundourou Lake is located in Western Attica. It is a lagoon with a mean depth of 1.5 m, laying upon the sea level, very close to the coasts of Elefsis Bay, and is separated from the sea by the Athens—Corinth National Highway [40]. Its water is brackish, also receiving freshwater. The lake was artificially created in antiquity when the construction of a coastal embankment blocked the passage of waters of adjacent springs towards the sea. Koumoundourou Lake has been declared as a protected archaeological site with a 50 m protection zone surrounding the lagoon (Official Journal of the Hellenic Republic 5/B/8.1.1974) and an Area of Outstanding Natural Beauty (site code: AT2011014), while it is included in MedWet wetland database (site code: GR300318000). However, its position nearby the industrial area of Skaramangas—Aspropyrgos has resulted in a highly polluted lake system. Demonstration of the ‘Chlα’ and ‘HM and Sampling’ USVs was performed in May 2019 at Koumoundourou Lake, collecting more than 30,000 datapoints of physicochemical parameters, covering almost its entire surface and three nearshore stations (demo, pumping and dam stations).

#### 2.4.2. Acheloos River

Acheloos River is the second longest river in Greece (Figure 3). Its total length, extending from its sources in the mountains of Pindos (Mount Lamkos or Peristeri) to its estuary at the border with the Ionian Sea (west of Aetoliko-Mesolongi lagoons) is 255 km, while its total catchment area occupies a surface of 6468 km^2^, with a maximum altitude of 2470 m [41]. According to the typology of the rivers of Greece [42,43], the study section is classified as type R-M3 (large rivers with mixed geology and highly seasonal flow), characterized by good chemical status [44]. Upstream of the study area, three dams constituting the Acheloos hydro-system scheme have been constructed to meet irrigation, hydropower, domestic and flood control needs. In September 2019, water quality monitoring was carried out in Acheloos River using the ‘Chlα’ USV. More than 10,000 datapoints of physico-chemical parameters, along a 2.5 km route upstream and downstream the river, from the Neochori to Katochi bridge, were collected.

#### 2.4.3. Kifissos River

Kifissos River is the principal river of Attica region, with its lower part flowing in parallel or below a highway, before reaching Faliro Bay within the Saronic Gulf. Kifissos is classified as river type R-M2 (medium rivers with mixed geology and highly seasonal flow), being the largest river of Attica Basin [44]. It has a length of 29 km, of which 14 km are within an urban area. Its catchment area is 420 km^2^, flowing through the central-western part of the Attica prefecture. The river is the main rainwater recipient of Attica. The floods that have occurred since ancient times, as well as the continuous expansion of the city limits, have led to structural interventions of the river. In July 2020, monitoring of the water quality of Kifissos River was carried out with the ‘Chlα’ USV and the research team collected approximately 15,000 datapoints of physico-chemical parameters along a 2 km stretch downstream of the river, from its mouth at Athens Marina to Piraeus Avenue (Moschato area).

#### 2.4.4. Asopos River

Asopos is classified as river type R-M2 (medium rivers with mixed geology and highly seasonal flow), with a length of 64 km, flowing along the borders of Boeotia and Attica Prefectures [44]. It originates from the Kithaironas mountain and empties into the southern Evoikos Gulf at the coastal area of Chalkoutsi town. Within the catchment area of Asopos, populated with approximately 70,000 inhabitants, very intense industrial and agricultural activity has been developed, leading to its consideration as one of the most polluted streams of Greece. Environmental pollution in Asopos waters became known in August 2007 when measurements by the General State Laboratory and other accredited laboratories detected the presence, among others, of hexavalent chromium in the water table of the area [41,45]. Monitoring of water quality at the mouth of Asopos River in Chalkoutsi was carried out in October 2019. The ‘HM and Sampling’ USV collected data of physicochemical parameters and heavy metals (copper and lead).

#### 2.4.5. Industrial effluents

The Metamorfosis Wastewater and Septic Sewage Treatment Plant (Metamorfosis WWTP) has been in operation since the 1980s in Greece, serving the purpose of treating both septic sewage and municipal wastewater. Situated in Athens, it holds the distinction of being the sole facility capable of receiving and treating septic sewage from areas lacking a sewerage system. This pivotal function has led to the cessation of uncontrolled sewage discharges into streams, thereby achieving a significant milestone in the protection of surface and ground waters. Two industrial effluent samples of varying origins were obtained and subjected to testing through a simulation operation conducted by the ‘HM and Sampling’ USV at the EYDAP laboratory.

## 3. Results and Discussion

Given the diverse nature of the study areas—comprising both a lake and multiple rivers—it is likely that the data collected from USVs and potentially integrated with telemetric stations would offer a comprehensive understanding of water quality dynamics across different water bodies. This comprehensive approach enables the identification of specific issues, trends or variations in water quality parameters across these varied environments.

### 3.1. ‘Chlα’ USV Campaigns for Physicochemical Parameters in Greece

#### 3.1.1. Chlorophyll α 

**Figure 4:**
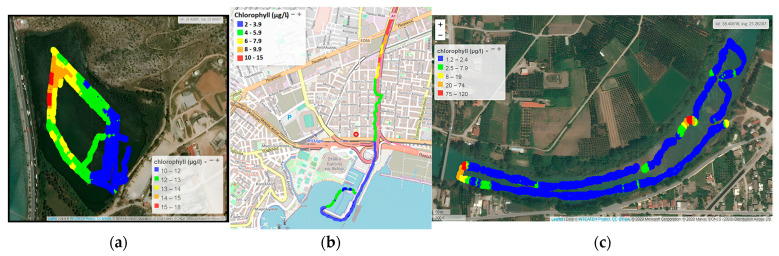
Chlα by the Waquin application of INTCATCH in (**a**) Koumoundourou Lake, (**b**) Kifissos river and (**c**) Acheloos river.

Chlorophyll α levels in Koumoundourou lake ranged from 10.0 to 17.5 μg L^−1^, being higher along the northeast part of the lake (Figure 4a), due to water circulation and groundwater influx. Regarding the Kifissos estuary, Chlα concentrations varied between 2.0 and 15 μg L^−1^, with an average value of 5.7 µg L^−1^ (Figure 4b). The significantly higher Chlα concentrations, reaching 15 μg L^−1^, measured upstream nearby Piraeus Avenue and the Piraeus urban railway station (Figure 4b), are attributed to organic matter accumulation during the inflow of the sea front upstream of the river bed. In the case of Acheloos river, Chlα levels ranged from 1.2 to 120 μg L^−1^ with a mean value of 5.2 μg L^−1^ (Figure 4c), significantly exceeded by the values detected downstream, nearby the Katochi bridge (120 μg L^−1^) and opposite to Neochori village (80 μg L^−1^), due to plant accumulation within river bed meanders and the formation of shallow reefs or micro-lakes.

The employment of USVs is characterized by several advantages, permitting the collection of data which cover a relatively large area, albeit within a small time scale. It therefore makes feasible the evaluation of potential trends, such as the decreasing one characterizing Chlα levels towards the Kifissos river estuary and the artificial bed close to Athens Marina, deriving through their spatial distribution. The results obtained for several parameters through USVs’ application may additionally be directly evaluated in the assessment of ecological quality, as in the case of Chlα measured herewith, which, according to the ECOFRAME system [46] classifying the ecological quality of shallow lake water, assigns Koumoundourou Lake within the “good” (11–20 μg L^−1^) category, aligning with earlier studies [47].

Furthermore, in previous studies [14,23], authors performed a comparative analysis of results from ‘Chlα’ USVs with satellite observations as well as with unmanned aerial vehicles (UAVs) in order to validate Chlα concentrations for another catchment in Greece (Lake Marathon). The analysis shows that the real-time results of ‘Chlα’ USVs in comparison with the evaluation of the images of the UAV and satellite observations are in accordance and can contribute to traditional monitoring programs of inland waters. In comparison with aerial and satellite observations for Chlα monitoring, ‘Chlα’ USVs offer distinct advantages. They provide real-time data transmission, enabling quick assessment and response to changes in Chlα concentrations. They also offer flexibility in data collection, especially in hard-to-reach or densely vegetated areas where aerial and satellite methods may be limited. Additionally, ‘Chlα’ USVs can cover smaller water bodies or areas with finer spatial resolution, complementing the broader coverage of aerial and satellite observations. However, aerial and satellite methods may offer wider coverage over larger areas in a shorter time frame, albeit with the need for image processing. Ultimately, the choice between USVs and aerial/satellite observations depends on factors such as the specific monitoring objectives, spatial resolution requirements and logistical considerations.

#### 3.1.2. Electrical Conductivity

**Figure 5:**
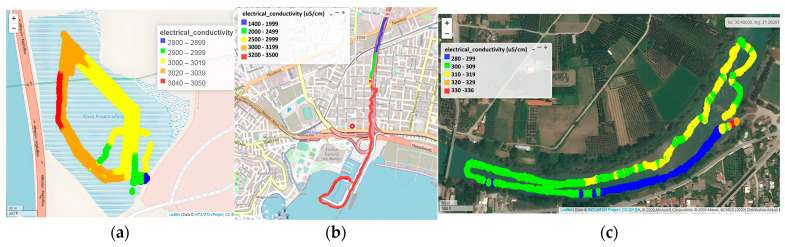
Electrical conductivity by the Waquin application of INTCATCH in (**a**) Koumoundourou Lake, (**b**) Kifissos river and (**c**) Acheloos river.

Electrical conductivity in Koumoundourou Lake ranged from 2800 to 3050 µS cm^−1^ (Figure 5a), with maximum values reported in the northeast, indicating a small inflow of seawater mixing through underground inlets. In the study area of the Kifissos estuary, the upper limit of 3500 μS cm^−1^ of the USV sensor was approached downstream of Arkadiou Street (beginning of red line), representing the IMBRIW station (Figure 5b). The EC range of 1400 to 3500 μS cm^−1^, reported during summer of 2000, indicated a significant inflow of the sea front, typical of brackish estuarine systems, in agreement with both recent and previous studies of the research groups of IMBRIW (HCMR) and other organizations within the specific area [47,48]. Within the studied part of the Acheloos plain, EC levels were relatively low, ranging from 281 to 336 μS cm^−1^ (mean value 308 μS cm^−1^). On the USV route from Neochori downstream to the Katochi bridge, two areas were distinguished in terms of EC, one within the range 280–299 μS cm^−1^, and another within 300–309 μS cm^−1^. Despite not being substantial, this difference is indicative of potential sensor discrimination (Figure 5c). The highest EC values measured at the shallow entry point of the USV near and upstream of Neochori (310–336 μS cm^−1^) are related to higher water temperature.

The assessed decreasing trend featuring EC in Koumoundourou Lake, reported by USV application, coincides with the findings recorded by HCMR studies from 1984 until today [47,49]. However, the comparison between data obtained by USVs and telemetric stations may indicate several differences, as in the case of Koumoundourou Lake, where the monitoring sensors of the telemetric station are installed close to the bottom, whereas unmanned boats operate superficially. The relatively low upper EC limit (3500 μS cm^−1^) of the USV sensor utilized herewith may be considered as a weakness when applied in estuarine areas, particularly during time periods of low river flow resulting in significant seawater intrusion upstream, leading to EC values reaching seawater salinity levels.

#### 3.1.3. Dissolved Oxygen

The concentration of DO is a reliable measure of the state and sustainability of an ecosystem. It is disrupted by either natural or human activities introducing an excess of nutrients into the water, leading to phytoplankton overgrowth and to anoxic phenomena, observed at areas close to the shore or nearby the bottom, characterized by organic matter accumulation. During summer, the concentration of DO on the epilimnion remains relatively high in temperate regions, owing to intense photosynthetic activity, aided by intense sunlight and atmospheric diffusion. Complying with pertinent environmental requirements, the application of USVs permits the reliable recording of DO geographical distribution over a wide area and in real time, constituting a significant advantage, particularly for sites that are difficult to reach. It simultaneously offers the possibility of identifying potential sources of environmental pressures related to discharges and surface runoff.

**Figure 6:**
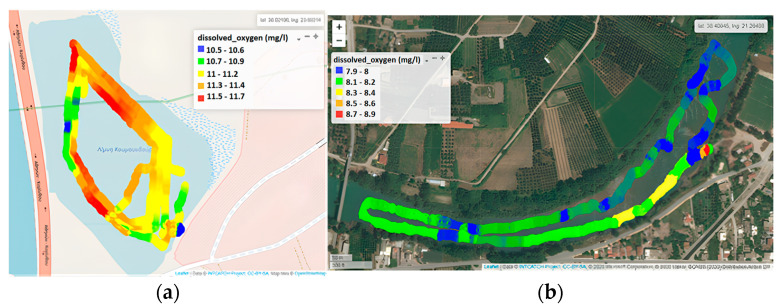
Dissolved Oxygen by the Waquin application of INTCATCH in (**a**) Koumoundourou Lake and (**b**) Acheloos river.

In Koumoundourou Lake, DO values ranged from 10.5 to 11.7 mg L^−1^ (Figure 6a), indicating well-oxygenated water compared to the study area of Acheloos River (Figure 6b), where the concentration of DO was reported at lower levels, ranging between 7.9 and 8.5 mg L^−1^, with an average value of 8.1 mg L^−1^. According to the limits applied for the characterization of different water quality classes set by the French SEQ System (Système d’évaluation de la qualité de l’eau des cours d’eau/SEQ-Eau), the ecological status of a river with a DO concentration exceeding 8.0 mg L^−1^ is considered ‘high’. Τhe spatial distribution of DO demonstrates higher concentrations (8.3 to 8.4 mg L^−1^) downstream of the river, nearby Neochori, attributed mainly to higher river velocity and water agitation. Simultaneously, several point intervals are recorded, mainly upstream of the river, with the concentration of DO remaining lower than 8.0 mg L^−1^ (Figure 6b, blue color illustration), consumed by the degradation of accumulated organic matter and/or other biological processes.

#### 3.1.4. pH

The pH of natural waters typically ranges between 4 and 9 units, with the range 6.5–8.5 being the most suitable for aquatic organisms. Water pH depends on various factors, including temperature, salinity (presence of sulfur anions, chlorine, cations of calcium, magnesium, etc.), carbon dioxide and oxygen levels, as well as the metabolic activity of aquatic organisms (such as photosynthesis and respiration) and organic substances’ chemical decomposition. Changes in pH are typically attributed to CO_2_ consumption in the epilimnion due to photosynthetic activity, resulting in a subsequent increase in pH. Recording of pH values through the application of USVs offers a significant amount of data, permitting coverage of wide areas, even remote, in both space and real time.

**Figure 7:**
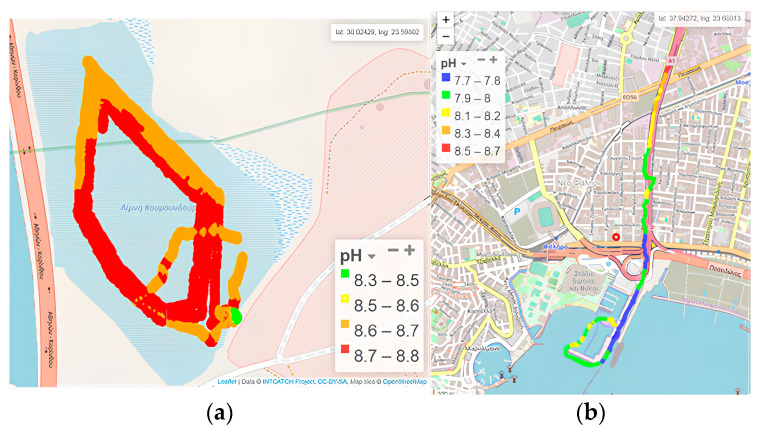
pH by the Waquin application of INTCATCH in (**a**) Koumoundourou Lake, (**b**) Kifissos river.

Within Koumoundourou Lake, pH was found to be alkaline, ranging from 8.6 to 8.7 (Figure 7a), with its spatial distribution demonstrating a slight decrease in the northeastern part, close to karst springs, attributed to fresh water inflow. According to ECOFRAME system [46], a pH range 6.0–10.0 (combined with Chlα) characterizes a ‘good quality’ system. The pH showed significant fluctuations in the study area of Kifissos River, ranging from 7.7 to 8.7 (Figure 7b), generally complying with the acceptable pH limits (6.5–8.5), except for specific high values measured. Higher pH values were determined upstream of the study area at the height of Piraeus Avenue, while a gradual decrease was observed towards the Kifissos estuary. The comparison with the corresponding values of time series recorded for the fixed station [48] of HCMR during July–August 2020 points out that pH values approximately equal to 9 appear frequently.

### 3.2. ‘HM and Sampling’ USV Campaigns in Greece

The ‘HM and Sampling’ USV was employed in Greece from January to October 2019, simultaneously in several demonstration activities and field campaigns. Within this framework, a fruitful collaboration was developed among EYDAP, HCMR-IMBRIW and the Laboratory of Environmental Chemistry (LEC) of the National and Kapodistrian University of Athens (NKUA), serving the perspective of contributing to the requirements of organizations and authorities for water quality, management and policy issues. The results obtained through the deployment of the HM and Sampling USV in lagoon systems and estuaries for metals measurements (in situ and in water samples) were combined (a) with those of corresponding measurements performed in natural samples obtained from the field and analyzed in the Laboratory of Environmental Chemistry of NKUA and in EYDAP laboratory, as well as (b) with relevant data previously reported both by LEC-NKUA and in pertinent literature.

Considering the lake systems examined, the values of total Pb_T_ and Cu_T_ determined herewith for Koumoundourou Lake, affected by industrial and urban activities, through the application of USVs were, respectively, equal to 6.2 and lower than 0.7 μg L^−1^ (Table 2). These results are comparable to those reported for the same area (Pb_Τ_ 0.038–3.49 µg L^−1^ and Cu_Τ_ 0.178 −3.22 µg L^−1^) by the Institute of Marine Biological Resources and Inland Waters of HCMR [47,49] (Table 2). Significantly higher values were previously provided for other Greek lakes [48,50,51] (Table 2), with Vistonis Lake of Northern Greece demonstrating the higher concentrations, supporting the considerable contribution of fertilizers and pesticides to Pb_T_ and Cu_T_ levels in lakes.

Regarding river systems, concentrations not exceeding 0.8 and 0.7 µg L^−1^ for Pb_T_ and Cu_T_, respectively, were reported in Asopos River, affected by industrial and agricultural activities (Table 2). These results are comparable to those recently reported for the Greek rivers Acheloos [41], Louros [41] and Asopos [52], affected mainly by agricultural pressures (Table 2). 

Dissolved metals present in aquatic systems form complexes with ligands deriving either from inorganic anions or dissolved organic matter, with only a small percentage of the metals remaining in the labile form, being available for detection using the SWASV method. Under this perspective, a comparison was performed among the results, obtained via applying the HM USV system, the technique of Inductively Coupled Plasma Optical Emission Spectrometry (ICP-OES) and the electrochemical method of Differential Pulse Anodic Stripping Voltammetry (DPASV), at three near shore stations of Koumoundourou Lake (demo, pumping and dam stations) (May 2019) and Asopos River estuary (October 2019) (Table 3). Field sampling was performed in parallel and the samples were analyzed by the ICP-OES method [53], in EYDAP accredited laboratory [54]. Electrochemical measurements of labile metals (Pb_L_ and Cu_L_) were carried out in LEC-NKUA by a voltametric instrument (μ-AUTOLAB, Eco-Chemie, The Netherlands) connected to a three-electrode cell (VA 663, Metrohm stand, Switzerland), with a static mercury drop electrode (SMDE) as the working electrode, the reference electrode Ag/AgCl (3 M KCl) and a carbon rod as the auxiliary electrode. The mode of Differential Pulse Anodic Stripping Voltammetry (DPASV) was applied. Prior to the field campaigns, calibration of the screen-printed sensor was performed in the laboratory, using mixed solutions containing a 1:4 Pb:Cu ratio. For a better representation of the concentration range of the studied metals in real surface waters, the calibration solutions were first prepared within a concentration range of 19–75 µg L^−1^ for Pb_L_ and of 75–300 µg L^−1^ for Cu_L_. The performance of the HM USV system was satisfactory considering the calibration procedure, with the measurements of real samples providing useful results subject to further discussion. Additional comparisons were carried out in two industrial effluent samples, where both Pb and Cu levels considerably exceeded those of natural samples.

The comparison of the results obtained, through the different analytical techniques applied, demonstrates the applicability of DPASV and ICP for the evaluation of the labile metal fractions and total metal concentrations, respectively. The considerable differences characterizing the results deriving for the same sampling areas employing SWASV and DPASV, applied under different experimental conditions for in situ and laboratory measurements, respectively, are attributed to the different detection window of these methods and mainly to the significantly higher LOD values of SWASV. The different working electrodes used, carbon paste screen-printed electrode (SPE) in SWASV and hanging mercury drop electrode (HMDE) in DPASV, as well as the different diffusion layers formed in each case, determine a different detection window and different limits of detection, hence imposing a detailed description of the experimental conditions applied.

The HM USV technique is so far suitable for cases of detecting point contamination by metals, rather than metal measurements in natural systems, due to a lack of sensitivity attributed to influences exerted from the water matrix and the concentration ratio of metals present. This becomes evident in the results obtained for field samples, in almost all cases of which Pb and Cu concentrations measured by SWASV were lower than the corresponding LOQs (Table 3). On the contrary, in cases of effluents samples, characterized by a particularly elevated Pb and Cu content, no significant differences are observed in Pb and Cu concentrations measured by SWASV and DPASV (Table 3). For this purpose, its sensitivity improvement, in order to respond to the low labile metal levels present in natural systems, becomes more than valuable for the direct detection of labile metal fractions in the field, avoiding any pretreatment of the samples.

The present work has set the initial basis for the application of the ‘HM and Sampling’ USV in the real time monitoring of metals. However, the significant differences featuring the results obtained for field samples with relatively low metal levels through the techniques of SWASV and DPASV denote a demand for improvement of the SWASV sensitivity and for achieving lower detection limits, in order to permit its application also to natural, unpolluted areas. Additional effort is required for the elaboration of such a perspective, which will exploit the valuable advantages for real time monitoring though direct in situ measurements, even in areas not easily accessible.

## 4. Conclusions

USVs enable a comprehensive and continuous monitoring approach, allowing for a detailed assessment of spatial and temporal variations. The application of two USV types was conducted to Greek catchments for the first time in the present work, clearly demonstrating their benefits in the process of water monitoring, together with their disadvantages imposing improvements in certain operational domains.

The employment of the ‘Chlα’ USV type, appropriate for the measurement of physicochemical parameters, in parallel with hydro-telemetric stations, achieves a thorough monitoring coverage both in space and real-time, providing a significantly higher amount of water quality data, even from hardly accessible sampling areas, without requiring any costly monitoring schemes. It also makes feasible the on-time raising of alerts in the attempt of elaborating action for protecting end users. The systematic, full-scale application of unmanned boats and hydro-telemetric stations could support investigative monitoring programs, representing a valid rapid tool/approach in cases of emergency (e.g., in relation to climate change events such as floods). However, since the sensors of the physicochemical parameters measured are installed close to the water surface, only data from that area are recorded, constituting a disadvantage of the USV system. In addition, the relatively low upper limit of, e.g., electrical conductivity (EC) featuring the corresponding USV sensor, requires improvement in order to become applicable to areas of naturally higher EC values such as estuaries.

Regarding the ‘HM and Sampling’ USV, it has been demonstrated that the developed and validated HM system [38] represents an innovative in-situ tool for investigative monitoring of aquatic environments, and it is considered fit-for-purpose for the detection of Cu and Pb from industrial discharges into small water body receivers and accidental pollution sources. However, certain improvements are necessary to overcome weaknesses related both to limited sensitivity and selectivity issues attributed to influences from the water matrix. The enhancement of the HM system for in situ detection of variations in the metal concentrations of surface water bodies also remain challenging to meet the limits of environmental quality standards (EQS) established by the WFD.

USVs provide real-time water quality reporting, crucial for effective decision-making and management, together with the collection of numerous discrete samples and the representative coverage of a study area (e.g., a whole lake) required for the chemical classification of a water body. Since the effectiveness of these vessels in rivers and lakes has been so far evaluated by a wide range of stakeholders involved, among others, in water quality, further development work is ongoing, aiming at enhancing sensitivity and minimizing or avoiding interferences by utilizing a fingerprinting approach. Additionally, designing USVs with enhanced environmental adaptability and user-friendly interfaces, and fostering collaboration with stakeholders for continuous feedback, are essential. Addressing these areas will help overcome existing limitations and unlock the full potential of USVs for more effective environmental management strategies.

## Figures and Tables

**Table 1:** Automatic telemetric stations of IMBRIW-HCMR.

Station	Latitude/Longitude	Altitude (m)	Installation Date	Website
Koumoundourou lake	38.0235° N/23.6018° E	0.83	28 March 2011	http://t.ly/p85TU (accessed on 20 April 2024)
Kifissos estuaries	37.9472° N/23.6727° E	3.35	15 July 2020	http://t.ly/UASuZ (accessed on 20 April 2024)

**Table 2:** Total dissolved lead (Pb_T_) and copper (Cu_T_) in Greek aquatic systems reported in literature.

Fresh Water System	Activity	Pb_T_ (µg L^−1^)	Cu_T_ (µg L^−1^)	Source
LAKES				
Koumoundourou	Industrial/Urban	6.2	<0.7	Present Study EYDAP (ICP-OES)
		0.038–3.49	0.178–3.22	Dimitriou, 2012 [47] Koussouris, 2014 [49]
Vegoritis	Fertilizer/pesticide	1.2–24.2	0.7–15.2	Zacharias, 2002 [50]
Mikri Prespa		0.2	14.4	
Koronia		36.8	3.7–21.8	
Vistonis		58.4	43.2	
Kastoria		31.1	6.6–19.4	
Doirani		22.3	9.6–12.4	
RIVERS				
Asopos	Industrial/Agricultural	<0.8	<0.7	Present Study EYDAP (ICP-OES)
Acheloos	Agricultural	0.07–2.85	0.01–5.40	Skoulikidis, 2018 [41]
Louros	Agricultural/Urban	0.05–0.48	0.24–0.60	
Asopos	Industrial/Agricultural	0.120–1.42	0.350–3.25	Botsou, 2007 [52]

**Table 3:** Comparison of results obtained by the SWASV method (HM USV), DPASV method (LEC, NKUA) and ICP-OES method (EYDAP) for total and labile Pb_L_ and Cu_L_, for Lake Koumoundourou (02/07/19), Asopos River and industrial effluents samples. Values in parentheses are <LOQ.

Metal	^1^Pb_L_ (µg L^−1^)	^2^Pb_L_ (µg L^−1^)	^3^Pb_T_ (µg L^−1^)	^4^Cu_L_ (µg L^−1^)	^5^Cu_L_ (µg L^−1^)	^6^Cu_T_ (µg L^−1^)
Sample Name/Method	HM USV (SWASV)	LEC (DPASV)	EYDAP (ICP-OES)	HM USV (SWASV)	LEC (DPASV)	EYDAP (ICP-OES)
Demo station Lake Koumoundourou	(8.1)	0.5	<0.8	(13)	0.8–1.0	<0.7
	(12)			(7.8)		
	16			(8.8)		
Pumping station Lake Koumoundourou	18	<0.1	6.2	(18)	<0.1	<0.7
	(8.5)			<7		
	20			27		
Dam station Lake Koumoundourou	(12)	<0.1	<0.8	<7	<0.1	<0.7
	(8.1)					
	<4					
Asopos River Estuary	(10)	<0.1	<0.8	<7	0.8–1.0	<0.7
	(7.6)			<7		
	(10.8)			(13)		
	(10.4)			<7		
Effluent 1	54	49	86	41	37	120
Effluent 2	71	65	116	62	60	185

^1^HM USV_Pb LOD_ = 4 μg L^−1^; ^2^DPASV_Pb LOD_ = 0.1 μg L^−1^; ^3^ICP-OES_Pb LOD_ = 0.8 μg L^−1^; ^4^HM USV_Cu LOD_ = 7 μg L^−1^; ^5^DPASV_Cu LOD_ = 0.1 μg L^−1^; ^6^ICP-OES_Cu LOD_ = 0.7 μg L^−1^.

## Data Availability

Data are contained within the article.
